# Differential Regulation of Toll-Like Receptor-Mediated Cytokine Production by Unfolded Protein Response

**DOI:** 10.1155/2018/9827312

**Published:** 2018-04-24

**Authors:** Sena Kim, Yeonsoo Joe, Young-Joon Surh, Hun Taeg Chung

**Affiliations:** ^1^School of Biological Sciences, University of Ulsan, Ulsan 44610, Republic of Korea; ^2^Tumor Microenvironment Global Core Research Center and Research Institute of Pharmaceutical Sciences, College of Pharmacy, Seoul National University, Seoul 08733, Republic of Korea

## Abstract

The ability of the host immune response is largely mediated by the proinflammatory cytokine production. Physiological and pathological conditions of endoplasmic reticulum (ER) trigger unfolded protein response and contribute to the development or pathology of inflammatory diseases. Under ER stress, unfolded protein response (UPR) signaling pathways participate in upregulating inflammatory cytokine production via NF-kappaB, MAPK, and GSK-3*β*. Moreover, it has been suggested that ER stress crosstalks with toll-like receptor (TLR) signaling pathway to promote the production of proinflammatory cytokines. In addition, TLR stimulation can lead to UPR activation to promote inflammation. In this review, we will cover how proinflammatory cytokine production by UPR signaling can be induced or amplified in the presence or absence of TLR activation.

## 1. Introduction

**Figure 1:**
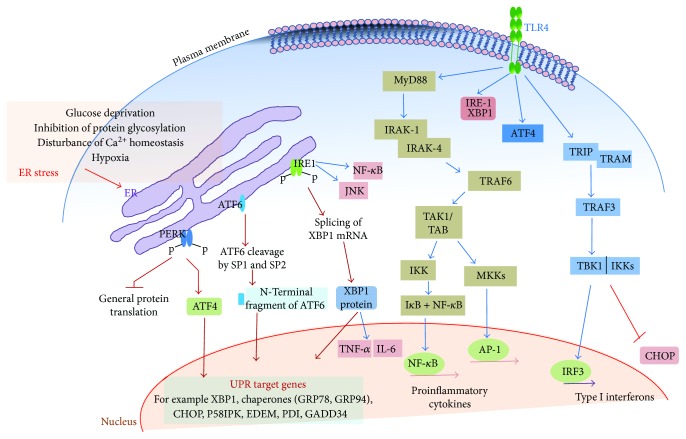
Interactions of ER stress proteins and cell surface TLR signaling pathways. Phosphorylated IRE1*α* interacts via TRAF2 with IKK complex and with ASK and JNK. Thus, IRE1*α* can activate NF-kappaB and AP-1. On the other hand, TLRs stimulate IRE1*α*-mediated splicing of XBP1 mRNA encoding various cytokines including TNF-*α* and IL-6. In addition, PERK-mediated translational inhibition leads to a shutdown of de novo synthesis of I*κ*B and thus leads to the activation of NF-kappaB. PERK and ATF6 induce ERK, p38 and JNK activation, resulting in increased expression of IL-6 and IL-8. ATF6 also activates NF-kappaB via phosphorylation of the Akt. ATF4 is involved in the expression of type I interferon and that of IL-23 via activation of GADD34 and CHOP, respectively. TLR4 tightly controls the ATF4-CHOP branch and prevents the induction of CHOP expression in macrophages via activating TRIF.

The endoplasmic reticulum (ER) is a crucial site involved in maintaining cellular functions, such as synthesis, modification, releases and translocation of proteins, biosynthesis of steroids, cholesterol and other lipids, metabolism of carbohydrates, and storage of calcium [1–3]. Numerous physiological and pathological conditions including imbalance in the ER folding capacity, accumulation of misfolded proteins, altered cellular metabolism, hypoxia, oxidative stress, infection, disruption of ER calcium ion balance, or N-linked glycosylation, can trigger the ER stress and initiate the unfolded protein response (UPR) to restore ER homeostasis and ensure cell survival [2, 4]. However, if the ER stress cannot be resolved, the UPR will initiate ER stress-associated programmed cell death to protect the organism by removing the stressed cells. When ER stress occurred in a cell, three individual ER-resident transmembrane branches of UPR begin with the dissociation from ER chaperon bip/grp78, followed by homodimerization and autophosphorylation of protein kinase RNA-like endoplasmic reticulum kinase (PERK) and inositol-requiring enzyme 1*α* (IRE1*α*) to activate the cytoplasmic kinase domains [5, 6]. In contrast, activating transcription factor 6 (ATF6) is translocated to the Golgi apparatus and then activated via proteolytic cleavage [7]. Activated PERK phosphorylates eIF2*α*, which transiently attenuates global mRNA translation, therefore reducing protein flux into the ER. Interestingly, certain mRNAs contain small ORFs in their 5′UTR such as activating transcription factor 4 (ATF4) can escape inhibition of translation. As a sustained translational inhibition is not compatible with cell survival, ATF4 induces GADD34, a regulatory subunit of protein phosphatase (PP1) acting as regulator of phosphorylation of eIF2*α*, to restore mRNA translation. ATF4 also induces the expression of the transcription factor DDIT3/CHOP, which is involved in ER stress-mediated apoptosis. IRE1*α* has functions of endoribonuclease and serine-threonine kinase. An endoribonuclease activity of IRE1*α* is a specific splicing of the XBP1 mRNA, which allow the translation of the spliced XBP1 (XBP1s) transcriptional factor. XBP1s has a major role in the induction of a wide variety of chaperones or proteins involved in protein-refolding, ER-associated degradation system, lipid metabolism, and proinflammatory responses [8]. After dissociation from bip, ATF6 is transported via coat protein COPII-covered vesicles to the Golgi compartment, where it undergoes intramembrane proteolysis by the Golgi enzyme site 1 protease (S1P) and S2P to produce active N-terminal fragment that translocate to the nucleus. Active N-terminal fragment pATF6-N directly induces the expressions of ER capacity and folding-related genes (such as GRP78, GRP94, GADD153, and XBP1) (Figure 1).

Toll-like receptor (TLRs) can recognize pathogen-associated molecular patterns (PAMPs) and danger-associated molecular patterns (DAMPs) and induce TLR-mediated intracellular signaling cascades to eliminate the pathogens through the production of proinflammatory cytokines including TNF-*α*, IL-6, IL-1*β*, and IL-8, but its uncontrolled activation can damage the host [9]. Sustained proinflammatory cytokine productions often contribute to the development of many inflammatory and autoimmune diseases. Upon ligands binding to TLRs, TLRs recruit adaptor proteins, myeloid differentiation primary response 88 (MyD88), and/or TIR domain-containing adapter-inducing interferon-*β* (TRIF) and transduce signals through interleukin-1 receptor-associated kinase (IRAK), TNF receptor-associated factor (TRAF), TGF-*β*-activated kinase 1 (TAK1), and receptor-interacting protein 1 (RIP1). In general, the MyD88-dependent response mediates the induction of proinflammatory cytokine, whereas the TRIF-dependent response mediates the induction of type 1 IFN response. TRAF3 interacts with both MyD88 and TRIF, but it differentially regulates MAPK signaling pathway and type I IFN signaling pathway, respectively [10]. TLR2 and 4 activation elicit TRAF3 ubiquitination which is the key to selective proinflammatory cytokine production through MAPK activation or type I IFN response through interferon regulatory factor 3 (IRF3). TRAF3 ubiquitination by TRAF6 and ubiquitin ligase cIAP1/2 resulted in MAPK activation and induction of proinflammatory cytokines in a MyD88-dependent manner [11]. Blockade of TRAF3 ubiquitination inhibits proinflammatory cytokine production through suppression of MAPK activation without anti-inflammatory cytokine production, IL-10, and type I IFN responses. Thus, MyD88-dependent signaling cascades by TLR stimulation result in the activation of NF-kappaB and MAPK, which are the central mediators of cytokine production (Figure 1).

ER stress has been shown to regulate proinflammatory cytokine production, which are mediated by TLR signaling cascade components such as NF-kappaB, MAPK, and GSK-3*β*. Studies have demonstrated that various metabolic syndromes are associated with chronic metabolic inflammation and impairment of ER function and established a link between inflammatory responses through the TLR signaling and ER stress response [12–15]. In recent studies on inflammatory diseases due to ER stress, inflammation was observed in models of tunicamycin-induced acute liver failure and various cancers [16, 17]. These reports may indicate the link between ER stress and inflammation, but the regulation of TLR-mediated proinflammatory cytokine production by UPR signaling is not completely understood. Ozcan et al. found a mechanism of ER stress-induced inflammation in white adipose tissue in high-fat diet-induced obese mice [18]. According to a study, it was provided that IRE1*α* aggravates inflammation and the phenotypes of obesity through the switching of M1-M2 macrophage polarization in a cell-autonomous manner [19, 20]. In addition, ER stress shares TLR-mediated signaling components with pro- and anti-inflammatory cytokine productions, leading to the activation of NF-kappaB and MAPKs. The XBP1 deletion or chemical chaperone treatment in macrophages alleviates proinflammatory cytokine production by LPS. In this review, we discuss ER stress and TLR-mediated signaling pathways for the regulation of proinflammatory cytokine production that linked ER stress to inflammation.

## 2. NF-KappaB and MAPK Activations in Inflammatory Cytokine Production by ER Stress

UPR is an important modulator in the induction of inflammatory cytokines, and UPR activation was shown to be sensitive to inflammation [21]. It has been well established that NF-kappaB activation is required for the induction of proinflammatory cytokines and has been linked to UPR [8, 22–24]. The PERK-induced inhibition of translation results in decreased translation of I*κ*B, which is a negative regulator of the NF-kappaB, therefore leading to greater activation and translocation of NF-kappaB transcription factor to the nucleus [25, 26]. A kinase activity of IRE1*α* directly triggers I*κ*B phosphorylation in a TRAF2-dependent manner, which results in the activation of NF-kappaB [27]. ATF6 also activates NF-kappaB via phosphorylation of the Akt [28]. These results indicate that UPR is sufficient to induce the proinflammatory mediator production such as IL-6, IL-1*β*, TNF-*α*, and IL-8 through NF-kappaB activation.

MAPKs (JNK, p38, and ERK) are also important inflammatory signaling molecules that induce inflammatory cytokines in response to ER stress [29, 30]. Chen et al. showed that HIV protease inhibitors (PIs) induce proinflammatory cytokines, TNF-*α*, and IL-6 through ER stress-mediated ERK activation, and these effects are diminished in CHOP knockout macrophages [31]. Thus, CHOP is responsible for HIV PI-induced ERK activation and proinflammatory cytokine production. The IRE1*α* can activate JNK in a TRAF2-dependent manner, leading to the increased expression of proinflammatory cytokines through activator protein 1 (AP1) [32]. Mijosek et al. demonstrated that ER stress induces phosphorylation of ERK, p38, and JNK through mainly PERK and ATF6 pathways in human primary bronchial epithelial cells resulting in increased expressions of IL-6 and IL-8 [33]. MAPK signaling pathways are involved in regulating the expression of inflammatory cytokines and ER stress-induced inflammatory cytokine productions that are also dependent upon the MAPK activation (Figure 1).

## 3. NOD1/NOD2 Signaling in Inflammatory Cytokine Production by ER Stress

TLRs and NOD-like receptors (NLRs) perform an important role in the recognition of microbial pathogens and damaged tissues. NOD signaling acts synergistically with TLR in proinflammatory cytokine production for the eradication of invading microbial pathogens. Fritz et al. demonstrated that TLR4 and NOD1 and NOD2 agonists lead to promote the production of proinflammatory cytokine in human monocytes and dendritic cells [34]. Roles of TLRs and NOD1/NOD2 on innate immune response have been proposed, but the NOD1/NOD2 signaling during ER stress-induced inflammation is not clear. Under ER stress condition, IRE1*α* branch in ER transmembrane is oligomerized and autophosphorylated. Activated IRE1*α* binds TRAF2 (TNF receptor-associated factor 2) to induce proinflammatory cytokine production via NF-kappaB signaling [35]. Yan and Liu showed that chemical ER stress inducers, thapsigargin and dithiothreitol, induced proinflammatory cytokine IL-6 in a NOD1/2-dependent manner. In addition, *Brucella abortus* strain RB51 induces IL-6 production through TRAF2-, NOD1/2-, and RIP2-dependent signaling pathway and could be abolished by ER stress inhibitor, tauroursodeoxycholic acid (TUDCA), or an IRE1*α* kinase inhibitor. Recently, Yan and Liu demonstrated that leucine-rich repeat kinase 2- (LRRK2-) dependent NOD1 activation by ER stress has a new positive regulator of RIP2 in inducing inflammatory cytokine in macrophages [36]. Therefore, these studies suggest that NOD1/2 activation with TLR is a new mechanism for ER stress-induced proinflammatory cytokines.

## 4. NLRP3 Inflammasome-Mediated IL-1*β* Maturation and Production by ER Stress

Inflammasome regulates IL-1*β* and IL-18 expression and maturation [37]. Studies have revealed that UPR-induced NF-kappaB activation and ROS generation are responsible for IL-1*β* and IL-18 secretions [38]. Kim et al. have shown that in murine and human macrophages, chemical ER stress inducers (tunicamycin and thapsigargin) and physiological ER stress inducers (palmitate and homocystein) induce pro-IL-1*β* and NACHT, leucine-rich repeat (LRR), and pyrin domain- (PYD-) containing protein 3 (NLRP3) expression and ER stress inducers-mediated ROS can trigger thioredoxin- (TRX-) interacting protein (TXNIP) dissociation from TRX to interaction with LRR and NACHT domain of NLRP3, which results in the assembly of the NLRP3 inflammasome to stimulate the activation of procaspase-1 and maturation of pro-IL-1*β* [39]. In addition, 4-phenylbutyrate- (4PBA-) mediated ER stress amelioration attenuates priming and maturation of IL-1*β* by ER stress inducers. Bronner et al. showed that ER stress can trigger NLRP3 inflammasome assembly in association with ROS generation from IRE1*α*-induced mitochondrial stress [40]. Treatment with TUDCA, a molecular chaperone that alleviates ER stress, and 4*μ*8C, IRE1*α* inhibitor that selectively inactivates RNase activity, decreased Bid truncation, caspase-2 and caspase-1 cleavages, and IL-1*β* production in RB51-infected or ER stress inducer-treated BMDM, but not LPS + ATP-treated BMDM [41]. *Brucella abortus* strain RB51 induces immune signaling through the induction of ER stress. Notably, ER stress-induced mitochondrial damage was required for the noncanonical NLRP3 inflammasome activation, which was mediated by IRE1*α*, caspase-2, Bid, and mitochondrial content release (mitochondrial-derived damage associated molecular patterns, mtDMAP) in an ASC-independent manner. Overall, ER stress regulates IL-1*β* and IL-18 production during chemical stress or microbial infection through canonical and noncanonical inflammasome activation.

## 5. GSK-3*β* in TLR- and ER Stress-Mediated Inflammatory Cytokine Production

Numerous studies have demonstrated that GSK-3*β* is a key regulator of inflammatory cytokine production in ER stress and TLR signaling [42, 43]. GSK-3*β* was found to strongly promote the production of proinflammatory cytokines by TLR-induced MyD88-dependent and MyD88-independent pathways, and the inhibition of GSK-3*β* was shown to protect the host from several inflammatory diseases such as colitis, arthritis, and sepsis-induced organ failure [43]. Studies by Martin et al. reported that stimulation of monocytes or peripheral blood mononuclear cells with TLR2, TLR4, TLR5, or TLP9 agonists induces substantial increases in IL-10 production while suppressing the release of proinflammatory cytokines after GSK-3*β* inhibition [42]. In addition, LPS-induced GSK-3*β* activation induces NF-kappaB activation and inhibits C/EBP*β*, CREB, and AP-1 transcriptional activities, diminishing the production of the anti-inflammatory cytokine, IL-10. Moreover, the inhibition of GSK-3*β* suppresses STAT3 activity resulting in reduced levels of IL-6 in LPS-treated mice and LPS-cultured primary glial cells [44, 45].

**Figure 2:**
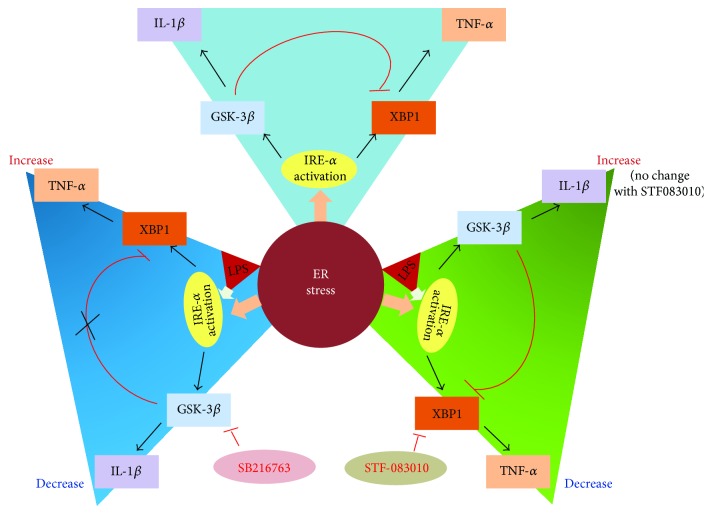
Interactions of downstream pathways of IRE1*α* for the transcriptional regulation of IL-1*β* and TNF-*α* in response to ER stress. ER stress-induced IRE1*α* activation differentially regulates IL-1*β* and TNF-*α* through GSK-3*β* activation and XBP1 splicing, respectively. IRE1*α*-mediated GSK-3*β* activation induces transcription of IL-1*β* but inhibits XBP1 splicing. Thus, SB216763, a GSK-3*β* inhibitor, selectively inhibits IL-1*β* gene expression and increases TNF-*α* production in response to ER stress. In contrast, IRE1*α*-mediated XBP1 activation results in the transcription of TNF-*α*. STF083010, IRE1*α* RNase inhibitor, suppresses TNF-*α* production without affecting IL-1*β* production. In addition, activation of XBP1 by ER stress inducers synergistically augments LPS-mediated TNF-*α* production. Likewise, GSK-*β* activation by ER stress inducer augments LPS-mediated IL-1*β*.

ER stress was also associated with increased activity of GSK-3*β* through the reduction of serine phosphorylation and promotion tyrosine phosphorylation by Akt inhibition and IRE1*α* activation, respectively [44, 46, 47]. The PI3K-Akt pathway has been shown to negatively regulate proinflammatory cytokine production through GSK-3*β* inactivation [44]. Guha and Mackman showed that the inhibition of PI3K-Akt pathway enhances LPS-induced TNF-*α* production through MAPKs (ERK, p38, and JNK) and nuclear translocation of NF-kappaB which induces the transactivation of p65 through GSK-3*β* activation [48]. Likewise, the inhibition of GSK-3*β* with LiCl reduces LPS-induced TNF-*α* in PBMCs and THP-1 monocytic cells, suggesting that GSK-3*β* positively regulates proinflammatory cytokine, TNF-*α*, through the reduction of transactivation of p65 without nuclear translocation of NF-kappaB. Therefore, the activation of GSK-3*β* by ER stress-mediated PI3K-Akt inhibition enhances the NF-kappaB-dependent inflammatory cytokine production. Recently, Kim et al. demonstrated that GSK-3*β* has a role in inducing inflammatory cytokine during ER stress [49]. The authors showed that the production of the proinflammatory cytokines IL-1*β* and IL-6 is triggered by the ER stress inducers thapsigargin and tunicamycin or LPS in a GSK-3*β*-dependent manner. However, TNF-*α* is regulated by IRE1*α*-mediated XBP1 splicing, independently of GSK-3*β* (Figure 2). These findings provide evidence for previously uncharacterized functions for GSK-3*β* on ER stress in the regulation of the immune response.

## 6. Crosstalk between UPR and TLR Signalings for Inflammatory Cytokine Production

Recent studies have shown that ER stress influences toll-like receptor-mediated intracellular signaling cascades involved in the activation of innate and adaptive immune responses [21, 50, 51]. Mahadevan et al. demonstrated that TLR4 KO BMDM treated with ER stress inducers shows decreased production of proinflammatory cytokines (IL-6, IL-23p19, and TNF-*α*) compared with WT BMDM [52]. On the other hand, TLR2 KO BMDM exposed to ER stress shows no decreased production of proinflammatory cytokine and increases the production of MIP-1*α*, MIP-1*β*, and MCP-1, suggesting that TLR2 may normally function as a negative regulator in response to ER stress-induced proinflammatory cytokine responses. On the other hand, Shimasaki et al. showed that ER stress enhances TLR2-dependent proinflammatory cytokine production (TNF-*α*, IL-8, and IL-6) through ATF4-mediated TLR2 upregulation [53]. These data suggest that ER stress-related proteins affect proinflammatory cytokine production through modulating TLR signaling.

Toll-like receptor-mediated activation of intracellular signaling pathways results in increased production of proinflammatory cytokines including TNF-*α*, IL-6, and IL-1*β*. Similarly, the activation of TLR ligands affects UPR signaling pathways. Microbial infection and TLR ligand treatment lead to the induction of UPR-related protein activation and gene expression, suggesting that UPR appears to closely interact with host immune response [54–56]. It was demonstrated that TLR2 and TLR4 activate IRE1*α*-XBP1 axis without PERK phosphorylation and ATF6 activation and promote the production of proinflammatory cytokines [57]. In addition, XBP1-deficient macrophages reduce IL-6 production in response to TLR agonists or infection with pathogen [8]. These evidences suggest that TLR-mediated IRE1*α* activation is intimately linked with inflammatory signaling pathways.

Several reports have shown that the concomitant treatment of TLR ligands and ER stress-inducing chemicals synergize the production of proinflammatory cytokines (IL-*β* and IL-6) [33, 57]. This amplified cytokine production can be regulated at the level of both the transcription and translation. According to some reports, this synergism depends on the p38, ERK, and GSK-3*β* activations. Mijosek et al. demonstrated that ER stress-mediated P38 and ERK activation is able to boost the production of inflammatory cytokines such as IL-6 and IL-8 in TLR- (TLR4, TLR3, and TLR5) stimulated airway epithelial cells. This synergic effect is mainly mediated by the activation of p38 and ERK via PERK and up-regulated p38 expression via ATF6 [33]. On the other hand, pharmacological activation of XBP1 or overexpressing XBP1s with the ER stress inducers synergistically augments TLR-mediated IL-6 and TNF-*α* productions. In addition, Kim et al. showed that ER stress inducer significantly augments LPS-induced proinflammatory cytokines (i.e., TNF-*α*, IL-1*β*, and IL-6) and GSK-3*β* activity in RAW264.7 macrophages and BMDM [49]. Rao et al. demonstrated that Kupffer cells isolated from 4-PBA-treated ischemic liver or ATF6-downregulated Kupffer cells from ischemic liver produce significantly less TNF-*α* and IL-6 after stimulation with LPS [58]. Thus, ATF6 activation in ischemic liver induces enhancement of proinflammatory cytokine production of macrophage in response to TLR4. Thus, the ER stress and TLR activation synergize the production of proinflammatory cytokines.

Some reports have shown that immune-enhancing drugs can boost the immune response to protect septic patients with the later immunosuppressive stage. Given that ER stress can restore cytokine production under endotoxin tolerance, it may be helpful to use ER stress induction to increase the cytokine production in the immune-depressed state. Thus, it is possible that ER stress under endotoxin tolerance condition might restore the immune capability to defend the host from infection. Indeed, ER stress inducers enhance clearance of bacteria through recovery of immune response under endotoxin tolerance condition (unpublished data). Thus, ER stress activation can be a novel therapeutic option via recovery of the immune response in patients with endotoxin tolerance.

## 7. Conclusion

The inflammatory response due to ER stress is frequently observed in the development of nonmalignant immunological disorders, such as rheumatoid arthritis and neurodegenerative diseases [12]. Evidences have shown that ER stress enhances TLR-induced intracellular cascades to produce proinflammatory cytokines (Table 1). However, more research is needed to understand the role of ER stress in host immune responses and to exploit this knowledge to design new drugs for patients with various inflammatory and metabolic diseases. Overall, this review emphasizes that the ER stress-induced inflammatory cytokine productions are shared with TLR-mediated signaling pathways and taking advantage of ER stress may be used as therapeutic option to prevent inflammatory diseases and protect secondary infection in septic patients through recovery of immune responses.

## Figures and Tables

**Table 1:** Differential regulation of proinflammatory cytokine production by UPR-TLR interaction.

Signaling components of TLR	Interacting UPR branches	Cytokines
NF-kappaB	PERK	IL-6, IL-1*β*, TNF-*α*, IL-8
TLR2	ATF4	IL-6, TNF-*α*, IL-8
ERK	CHOP	IL-6, TNF-*α*
NF-kappaB	IRE1*α*	IL-6, IL-1*β*, TNF-*α*, IL-8
TRAF2-NOD1/2		IL-6
TRAF2-GSK/JNK/p38		IL-6, TNF-*α*, IL-8, MCP-1
TRAF2-IKK		IL-6, TNF-*α*, IL-8, IL-2
TRAF6		IL-6, TNF-*α*
GSK-3*β*		IL-1*β*
NF-kappaB	ATF6	IL-6, IL-1*β*, TNF-*α*, IL-8
p38		IL-6,IL-8
